# Complementing a Clinical Trial With Human-Computer Interaction: Patients’ User Experience With Telehealth

**DOI:** 10.2196/humanfactors.9481

**Published:** 2019-06-06

**Authors:** Sakib Jalil, Trina Myers, Ian Atkinson, Muriel Soden

**Affiliations:** 1 Betty Irene Moore School of Nursing UCDavis Health University of California - Davis Sacramento, CA United States; 2 Information Technology James Cook University Townsville Australia; 3 eResearch Centre James Cook University Townsville Australia; 4 School of Medicine James Cook University Townsville Australia

**Keywords:** clinical user-experience evaluation, telehealth, type 2 diabetes, user experience, human-computer interaction, patient-centered, patient-technology interaction, eHealth

## Abstract

**Background:**

The use of telehealth to monitor patients from home is on the rise. Telehealth technology is evaluated in a clinical trial with measures of health outcomes and cost-effectiveness. However, what happens between a technology and the patients is not investigated during a clinical trial—the telehealth technology remains as a “black box.” Meanwhile, three decades of research in the discipline of human-computer interaction (HCI) presents design, implementation, and evaluation of technologies with a primary emphasis on users. HCI research has exposed the importance of user experience (UX) as an essential part of technology development and evaluation.

**Objective:**

This research investigates the UX of patients with type 2 diabetes mellitus (T2D) with a telehealth in-home monitoring device to manage T2D from home. We investigate how the UX during a clinical trial can be researched and what a clinical trial can learn from HCI research.

**Methods:**

We adopted an ethnographic philosophy and conducted a contextual inquiry due to time limitations followed by semistructured interviews of 9 T2D patients. We defined the method as Clinical User-experience Evaluation (CUE). The patients were enrolled in a telehealth clinical trial of T2D; however, this research was an independent study conducted by information technologists and health researchers for a user-centered evaluation of telehealth.

**Results:**

Key analytical findings were that patients valued the benefits of in-home monitoring, but the current device did not possess all functionalities that patients wanted. The results include patients’ experiences and emotions while using the device, patients’ perceived benefits of the device, and how patients domesticated the device. Further analysis showed the influence of the device on patients’ awareness, family involvement, and design implications for telehealth for T2D.

**Conclusions:**

HCI could complement telehealth clinical trials and uncover knowledge about T2D patients’ UX and future design implications. Through HCI we can look into the “black box” phenomenon of clinical trials and create patient-centered telehealth solutions.

## Introduction

### Background

Type 2 diabetes mellitus (T2D) is currently one of the world’s fastest-growing diseases; the prevalence of T2D rose from 171 million persons affected in 2000 to 415 million in 2015 worldwide [1]. The total annual global health expenditure for diabetes in 2015 was US $673 billion. The cost accounted for 12% of the world’s total health expenditure [1].

Treatments for T2D involve diet control, exercise, home blood glucose testing, and, in some cases, oral medication with or without insulin [2]. Effective individualized treatments may also incorporate psychosocial, lifestyle, and other medical interventions [3].

Technology-mediated treatments, such as telehealth, eHealth, mHealth to monitor patients from their homes, are on the increase with chronic diseases such as T2D. Telehealth is the use of information and communication technology (ICT) to provide clinical treatments over distances [4]. A common telehealth treatment for T2D is for patients to send regular blood glucose data to nurses or health care providers via phone, tablet, computer, Web-based system, videoconference, phone call, or short message service (SMS) text [5,6]. A nurse or health care provider is involved in T2D telehealth treatments continuously, while the technology intervention remains as a means of transferring data (eg, blood glucose, blood pressure) and facilitates the communication between patients and nurses for better management of T2D [5,6].

During evaluation through randomized clinical trials, telehealth technology is represented as a “black box.” Systematic reviews have shown that clinical trials assess “what went in” (eg, baseline measures) and “what came out” (eg, postintervention measures). “What happens inside the interventions” (eg, how patients felt about using the device and the development of the interventions not achieving a match between technology and context) is rarely a focus of attention in clinical trials [7,8]. For example, in a clinical trial of T2D, the long-term blood glucose HbA1c of patients at baseline is compared against HbA1c at the end of the trial. Improvements in HbA1c, along with additional health parameters, are data that the clinical researchers use to conclude whether a telehealth technology for T2D was effective or not.

Clinical trials do not investigate the relationship between the technology and effects of the use on patients as technology users, how patients interact with these technologies, or how patients feel when using these technologies [5,8]. However, the discipline of human-computer interaction (HCI) tends to be highly divergent in the choice of methods and approaches to understand humans and their interactions. A common practice in HCI is to understand user experience (UX) to design and develop a human-centered technology. UX refers to how a product behaves and is used by people in the real world [9].

We were interested in solving the “black box” phenomenon of a telehealth T2D clinical trial. We looked at six common methods (Table 1) of HCI to explore if we could use one or more of them during clinical trials to understand the UX of patients with T2D with telehealth.

Upon investigation of the six methods in Table 1, we concluded that there was no possibility to conduct a codesign, participatory design, lead user approach, or empathic design because these methods are conducted to create new solutions along with stakeholders. In a clinical trial, a device already in use is selected already by doctors, nurses, and stakeholders. Next, the effectiveness of the device is evaluated, and user-centered design methods are not practiced in a clinical trial. Therefore, we were only left with two options: applied ethnography and contextual design inspired by ethnography.

We adopted an ethnographic philosophy for this study to understand how the situation is in a clinical trial by moving the researchers into the users’ environment. Due to time and resource restrictions, we deduced to conduct a contextual inquiry and observations, followed by a semistructured interview, and finally another follow-up via survey. This HCI-inspired research method was named Clinical User-experience Evaluation (CUE) [6]. We wanted to conduct an independent study from an HCI perspective; therefore, we went through a process of defining CUE and its differences from the clinical trial. This paper presents the results of the UX of patients with T2D with telehealth.

**Table 1:** The 6 dominant human-computer interaction methods.

Method	Key feature	Research orientation
Applied ethnography [10]	Long-term immersive fieldwork; observation combined with participation	Researcher moves into users’ world
Contextual design [11]	An ethnographic approach to finding the specific needs of users in a work situation; provides 8 methodological steps	Researcher moves into users’ world
Empathic design [12]	Draws on information about the user and her everyday life, and includes inspiration for design and empathy, or “a feel” for the user	Researcher moves into users’ world
Participatory design [13]	Users who will be using a system are given a role in the design, evaluation, and implementation of the system	Users brought into the researcher’s world
Co-design [14]	May invite users and other people who do not yet know each other; design a product for a mass market or nonwork contexts	Users brought into the researcher’s world
Lead user approach [15]	Brings innovative users together, as many ideas of new products or services originate in the minds and hands of them and not from professional researchers and designers	Users brought into the researcher’s world

### Research Objective

The research objective was to investigate how to discover patients’ UX in telehealth, eHealth, and mHealth in a clinical trial. To pursue the research objective, we answered the following three questions with the CUE:

1. What happens at the patient’s home during the use of the telehealth device?
2. How do patients feel while using the telehealth device?
3. Which function(s) and designs of the device satisfies/ dissatisfies the patients?

## Methods

### Research Method

**Figure 1:**
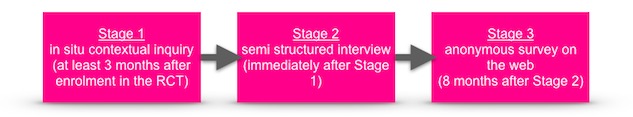
The Clinical User-experience Evaluation (CUE) methodology.

An investigation through meta-synthesis conducted in 2014 of past clinical trials of telehealth T2D concluded that there is a need for new practices that could capture the experience of users (patients) in a clinical trial [6]. Therefore, we created the CUE. The CUE consisted of three stages (Figure 1). Stage one was a contextual inquiry performed in situ at a patient’s home. During this stage, a patient used the device with the think-aloud method as one researcher as the observer took notes. This contextual inquiry was conducted during a patient’s regularly scheduled time for using the device, in the patient’s home. Stage two was a semistructured qualitative inquiry into the patients’ experience and expectations, the questions that developed during stage one, and anything extra the patient wanted to talk about. The interview took place directly after stage one on the same day, while perceptions were still fresh in the mind of the user. Stage three was an anonymous survey to follow-up with patients the findings from the first two stages and if there were any changes in the use of the device. This was conducted 8 months after stage two. The researchers were ICT researchers from James Cook University Townsville (Queensland, Australia) who had no involvement with the clinical trial. Every participant was enrolled at least 3 months (12 weeks) into the clinical trial to avoid novelty effects.

During the application of the CUE, health professionals asked us (the HCI researchers) to articulate the contribution of CUE as opposed to a clinical trial, especially because the clinical trial is a 300-year-old methodology [16] used in medical science. The CUE protocol is compared to the clinical trial in Table 2 to show the differences. Because clinical trials are regulated protocols, this table supported us to convey the information to the team of health scientists (nurses and physicians).

**Table 2:** Differences between the Clinical User-experience Evaluation (CUE) and clinical trials.

Review criteria	CUE^a^	Clinical trial
Investigation aims	Investigates patients’ experience, understanding, feeling, and usage of a technology for health care	Investigates patients’ medical condition with an intervention that can be a drug or a technology
Outcome	To provide patient feedback about using the trial technologies and a guide for future improvement of the technology, including features that were lacking or nonexistent that would benefit the treatment process	To provide enough evidence for medical practitioners to make sound judgments
Sample size	A smaller sample population similar to HCI^b^ qualitative user evaluation is appropriate	Requires large sample population to provide substantial and robust evidence
Regulations	Tests interaction with a device without interfering in any medical protocols, there is no physical or psychological stress; conducted at the regular times a patient uses the technology as part of the overarching clinical trial	Rigorous form of testing that must follow HTA^c^ guidelines; clinical trials often include psychosocial analysis questionnaire
Investigator	Can be carried out by anyone working in the field of HCI with simple practice and observational skills	Carried out by medical staff or caregivers who have either medical credentials or training in health care and/or social work
Recruitment	Participants come from the clinical trial	Larger samples of volunteers are sought who have specific medical conditions
Ethics	Privacy of information is required, and the participant must provide written consent	Strong, regulated ethical process and abiding by HTA regulations

^a^CUE: Clinical User-experience Evaluation.

^b^HCI: human-computer interaction.

^c^HTA: Health Technology Assessment.

### Participants: Inclusion Criteria

The CUE was applied on a clinical trial that was conducted by Townsville-Mackay Medicare Locals in North Queensland, Australia [17]. A total of 210 patients were recruited in Townsville, Mackay, and Brisbane. Participants were referred by two nurses. The participants of the CUE were (1) enrolled in the clinical trial, (2) belonged in the intervention group (using the telehealth device), (3) diagnosed with T2D for at least 12 months, and (4) volunteered to participate in CUE.

### Participant Details

Participation in the CUE was voluntary. A total of 12 patients initially agreed to participate. However, three of them opted out of the CUE study because they were not available during the designated time frame. Nine patients participated in the CUE study. Five of them were considered part of the aging population with an age of at least 64 years, and four participants were within the age range of 50 to 63 years (Table 3). Participants were given pseudonyms that were incredibly different from the participants’ original names. In addition to the nine participants, five family members occasionally provided feedback. Of these five, only two family members permitted us to use their quotes.

**Table 3:** Participant details (N=9).

Participants pseudonyms	Sex	Age (years)	Computer use (hours/week)	Time in clinical trial (months)	Time since diagnosed with T2D (years)
Uma	Female	74	0	5	>12
Zach	Male	70	70	8	>10
Yanicka	Female	68	20	6	7
Vince	Male	66	20	6	>10
Bill	Male	64	4	5	20
Heidi	Female	60	2	5	25
Serena	Female	55	12	3	2
Pete	Male	53	2	6	1
Ted	Male	52	60	6	2

**Figure 2:**
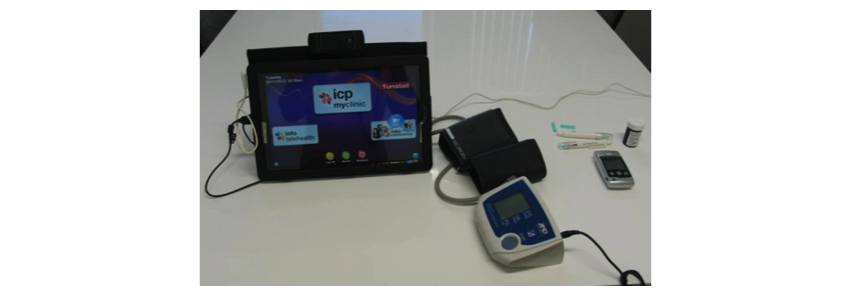
The in-home monitoring device of the clinical trial: a tablet PC, sphygmomanometer, and glucometer.

### Equipment

Participants used a tablet computer with an 11-inch screen, an automatic glucometer, and an automatic sphygmomanometer (Figure 2). The device had a touchscreen interface and was a single-user system. A regular patient session entailed a patient turning on the tablet and waiting to log in automatically. The patient then looked at the scheduled blood glucose and blood pressure test that was arranged by the nurse. The patient pricked a finger to get a drop of blood and put it on a strip for a blood glucose reading. The strip was then placed in the glucometer. To get the blood pressure measurement, the patient put an arm in the sphygmomanometer, which automatically took the reading.

### Data Analysis

Interviews and contextual inquiry sessions were audio recorded. The recordings were transcribed, and the notes and data from the contextual inquiry were analyzed using the contextual design methodology (Multimedia Appendix 1). The semistructured interviews were analyzed with thematic content analysis; NVivo 10 software was used to manage the analysis process.

## Results

The results showed two themes: (1) the current design and how that fits with the patients’ needs, and (2) the patients’ experience of using the device depicted through their feelings and perceptions.

### Current Design: Technology-User Fit

#### Placement of the Device

We found that patients placed the device in different parts of their homes (Table 4). The patients chose to place the device in four locations: living room (n=4), bedroom (n=2), study room (n=2), and patio (n=1). Reasons mentioned were internet or phone socket availability (n=3), convenience (n=4), comfort (n=1), and self-motivation (n=1) regarding their choice of device placement.

**Figure 3:**
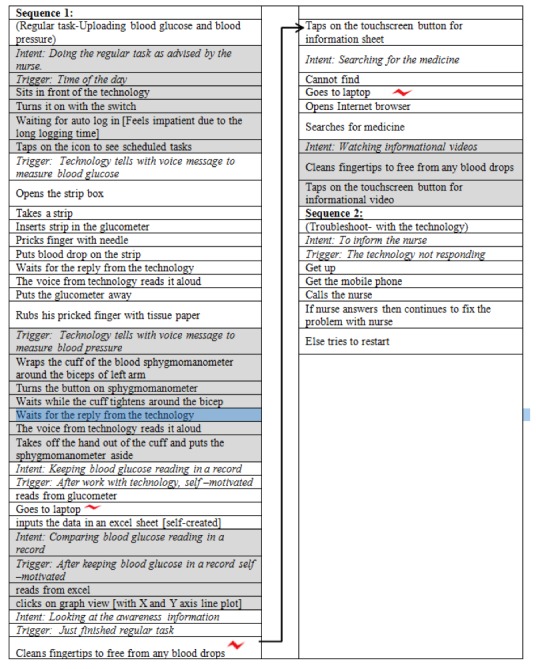
Example of one sequence diagram that shows breaks of patient.

Data from the contextual inquiry was first analyzed through four steps of the contextual design method (see Multimedia Appendix 1). The results from one exemplary case, Zach’s sequence model, showed that he had three breaks noted with a red mark (Figure 3). The breaks were (1) to save data because previous readings were not saved for the patients, only for the nurses, (2) to clean his fingertips after the blood test to continue with the touchscreen, and (3) to use the internet on a different device because the telehealth device did not have names of all medications.

**Table 4:** Placement of the device in the patients’ homes (N=9).

Reason	Location, n				Total for reason, n
	Living room	Study room	Bedroom	Patio	
Internet socket	2	1	—^a^	—	3
Comfort	1	—	—	—	1
Convenience	1	1	1	1	4
Self-motivation	—	—	1	—	1
Total in each room	4	2	2	1	

^a^Room-reason not selected.

#### Lack of Wireless Capability

The device only functioned with wired internet that had to be connected through a cable through the telephone port in a patient’s house. Heidi, Serena, and Uma mentioned that having wires was a problem of the device:

Apart from when you gotta be home two hours after eating to do it can be a little bit difficult like, “Oh my God I have gotta get home,” so, I mean, time-wise that’s it if I am not gonna be at home.

Heidi

When Uma traveled, she used a separate glucometer and would keep her blood glucose readings in a diary. She would later come home and update her nurses about the data. However, the device did not allow users to record data manually:

I can take this [her own glucometer that she bought] with me, I can’t do the blood pressure, I take this with me and do the blood sugar and then put it down in a book.

Uma

#### Undesirable Experience From Sphygmomanometer

Every patient criticized the sphygmomanometer. It was difficult to use. It also gave uncomfortable experiences:

The blood pressure cuff I have more difficulty with. I put it here where my doctor would put it. It repumps, and it takes ages to do it. It marks my arm.

Yanicka

Yanicka complained of physical pain around her arm from the device. She stated that this pain was more than other sphygmomanometers that she had used in doctor visits.

#### Lack of Visual Data

In the current system, each time the patients conducted a test, they were presented with instant data on their blood pressure and blood glucose levels. However, when the patients conducted the next scheduled test, they could not see the previous data. For example, if a patient did a test in the morning and one in the evening, they were unable to compare the readings, because the earlier test was not available. Patients expressed their desire and the importance to see the previous data to help them know if they were doing better or worse in terms of their blood glucose:

I know it does it here [glucometer], but it would be good to see every day’s. But it doesn´t show you. Like last week I might have been 5.5 and this week I am 7.5. Why? Why am I? Then I would do exactly the same things that I did last week.

Bill

Vince and his wife also mentioned the adjustment of insulin, similar to Heidi. They said that while Vince took insulin and was adjusting the dosage of the insulin, they would prefer to see a day-by-day comparison of Vince’s blood sugar in a graph:

It would be much better if he could just push a button and see the last three weeks of his readings.

Coz he is adjusting his insulin and he needs to know—all the time.

Vince’s wife

Probably I would like to see a graph of my results, more often. Like even once a month would be good to show it on a graph. How my results are going, because you just see number every day, but you want to know your ups and downs, and you want to know using that computer why my diabetes goes higher, I know the reason now why it goes higher, before I didn´t know the reasons. But now I do. And it’s just the difference the food that I have eaten, and the foods prepared, and I have found that because I am monitoring my blood glucose carefully.

Pete

Zach stated that graphs are a great tool to compare trends. Zach was very particular about using a progressive graph. He also commented that much research is required on how to show the blood pressure and the blood glucose level in the graph:

There is nothing like graphs to see trends. They have to display in a sensible way, if that makes sense. I will be thinking that a progressive graph will do it.

Zach

#### Lack of Medication Name

Yanicka stated that the medication that she was taking was not included in the information sheet listed on the device. This meant that the database did not contain a full list of all possible diabetes medications that the patients in this clinical trial were using. This necessitated Yanicka using another computer to locate information about the medication that was prescribed for her:

To see my change of insulin and I couldn’t find on here, so I went back through here with my computer and internet. My medicine is also here...and insulin is not there, but I looked that up at the computer. Not everyone has that. When I want to see what that thing do I check it up here. I don’t ever touch the unit because it automatically shuts down. It’s simple as that, quite easy to use. Bit challenging at the beginning.

Yanicka

Zach reported the same problem—his medication was missing from the available information sheet on the device.

#### Mismatch With Life Due to Immobility of the Device

The device currently works only with internet cables. All the patients stated that a mobile unit would have been much more suitable than the current device. Uma stated that she could not carry the device. So she carried a different glucometer to keep the data for her records:

I can take this with me; I can’t do the blood pressure, I take this with me and do the blood sugar and then put it down in a book.

Uma

#### Glucometer Discomfort and Pain

When a patient uses a glucometer, a small drop of blood is obtained by pricking the skin with a lancet. The drop of blood is placed on a disposable test strip that the meter reads and uses to calculate the blood glucose level. Slight discomfort is experienced when the lancet pricks the skin of the finger. However, T2D patients use a glucometer frequently, often more than once a day. Some of the patients in this clinical trial mentioned the discomfort and pain from the glucometer. Ted stated that after frequent use over a long period, his finger feels bruised:

Problem I see with this is you have to prick your finger every time you use it. It’s not that bad but after a while you are bruising your fingertips sore, so in that respect I guess it’s not really something that one looks forwards to going and doing.

Ted

Every other patient also felt the pain and complained of being hurt. As a remedy, Zach was interested to see what the scientists come up with in the future. Ted also mentioned that he wants science to advance in such a way that a chip can be inserted and left in a human body so that it will transmit continuous readings to the machine. In this way, Ted thinks, bruising and pain may be avoided.

### Feelings and Perceptions

Patients used words such as “motivation,” “accountability,” “safety net,” “habit,” and “awareness” while they expressed their frustrations with the telehealth device.

#### Motivation

Two participants, Vince and Heidi, mentioned that using the device motivated them to manage their diabetes:

And it’s good that they [nurses], that someone else is keeping an eye on you, back at office, nurses.

Vince

And it gives you just that extra push, you know?

Heidi

#### Build a Habit

Pete lives alone, and he stated that he had developed a habit from using the device for 6 months in the clinical trial. His habit was measuring his blood glucose and blood pressure early in the morning before he would engage in his daily life:

I think it’s a great benefit for me, I wish it probably could stay, and I would like to keep it. I don´t know how I am gonna go; I am obviously in the habit of doing it every morning now, I am gonna have it. It´s a habit now. So next week it’s gonna go, and I can still maintain the regimen that I am doing it now, you know.

Pete

#### Awareness

Enrollment in the clinical trial had made Serena aware of her well-being. The device would make her do things regularly. Serena called this being in a regimen where she had to regularly monitor and be aware of her blood glucose and her food. Serena’s son, who was one of the family members to permit his data to be used in this research, mentioned:

It’s more like a—there’s a regimen for every day 10 minutes before eating and after eating, she tastes it and morning, afternoon—it’s 10 minutes or 5 minutes—doesn’t affect much. But it improved her overall awareness.

Serena’s son

Vince stated that after he had looked at the results, he felt more aware and accountable, which made him want to use the device more:

It [the device] makes you, wanna do it [the blood glucose reading].

Vince

Heidi compared the use of the telehealth device with quitting smoking. In quit-smoking programs, people are typically encouraged to call a back-end, or a buddy, each time they have the urge to smoke. Heidi found using the device a similar experience as it makes her do the one extra step that she needs to take:

You know when you haven’t done this for a week, and oh you should do it. It’s like quitting smoking; you know that you have to ring up somebody every time you have to ring up. So, it’s that extra incentive you know.

Heidi

#### Feel Safe

Daily monitoring provided safety and comfort to the patients. In the case of Vince, daily monitoring made his wife feel safe that someone was watching over him:

It’s sort of like a safety net. You know there’s someone in the background always watching and they will ring you up.

Vince’s wife

For Uma (a 74-year-old woman living alone), the device was not of interest. In Uma’s opinion, the use of the device provided the nurses with the data that they needed and that made her feel safe. Serena’s son stated that Serena’s enrollment in the trial and use of the device helped him to look after her.

#### Reduced Doctor Visits

Patients stated that they had fewer visits to the doctor during the time enrolled in the clinical trial. They indicated that they did not have to see a doctor every 3 months, which is the traditional treatment. Instead, they spoke with the nurse every 2 weeks, which decreased the doctor visits unless there was something urgent.

#### Frustrations

Patients had frustrations using the device due to slow responses and sometimes during unresponsive states. Even after participating in the clinical trial for more than 3 months, the patients often had problems with the device. For example, 74-year-old Uma, in her fifth month in the clinical trial, was very frustrated during the contextual inquiry. A portion of the transcript (from the second minute until the fifth minute) of Uma is as follows:

Uma: I don’t know what’s wrong with it; it suddenly slowed down.

Researcher: Did it slow down today or—?

Uma: No, it has been doing this for a few days. I was talking to the lady [Nurse1] on the phone and—come on.

Uma called “come on” to the device after being frustrated with the device for not responding to her touches.

Uma: I have to go through this every morning. It’s—aaah.

Uma ceaselessly showed frustration, sighed heavily with hand gestures toward the device, and talked to the device.

Uma: I don’t know whether it’s because it’s—aaaahhhhh.

more frustration

After the fifth minute, Uma was able to use the device after restarting it and being helped by the researcher.

#### Difficulty in Measurement of Blood Pressure

**Figure 4:**
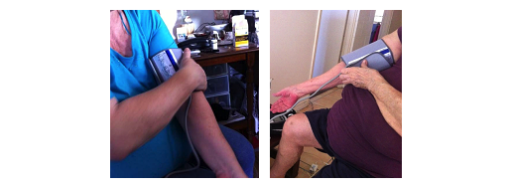
Heidi (left) and Uma (right) struggling with the sphygmomanometer.

All but one patient (Ted) complained about difficulty with the automatic sphygmomanometer because they had to use one arm to put the cuff around the other arm and then press a button on the device screen to start the automatic adjustment process (Figure 4). It was a very difficult process for any person to do this task alone. Heidi described it as: “It’s not really a one-man job.”

## Discussion

### Overview

All the patients were more than 3 months in the clinical trial. Yet, we saw frustrations during use of the device due to design—the responses and limitations. There were perceived benefits and promises if designed right. Even how the treatment was designed was influencing patients UX. For example, T2D patients had to measure their own blood pressure, which is not an easy task. Even the researchers could not measure blood pressure accurately with the same device during some practices.

### Design Implications for Future Telehealth for T2D

The patients wanted to see their own data meaningfully presented through graphs. And a wireless device was preferred due to mobility. Glucometer comfort, inclusion of all medication names, and wireless connectivity are essential for a device for T2D.

### Domestication of the Device

The patients treated the device like regular domestic technology. Stable and compelling routines at home influences the use of domestic technologies [18]. Therefore, considerations of people’s routine activities and contexts are essential to inform the design; otherwise, people end up excluding those technologies. Our results resemble Crabtree’s findings [19]; in domestic settings, the patients might have multiple other gadgets, and the telehealth device became one of them. Ted placed his device in his living room beside his reclining chair, which shows comfort as a reason. Other participants, such as Vince and Yanicka, chose their device locations based on convenience.

### Influence on Patients From the Use of the Technology

The study of how to design technology to motivate behavioral change has been of increased interest to researchers and industrial practitioners due to the widespread use of technology, such as computers, mobile phones, iPads, etc. Persuasive technology is “a computing system, device, or application designed to change a person’s attitude or behavior in a certain way” without using coercion or deception [20]. Additionally, technology is never neutral; it influences users in one way or another [21,22]. Persuasive technology is designed to target a specific behavioral change of the users intentionally. These study patients mentioned different levels of influence on their lives from the use of the device. Heidi said she received extra motivation from this device to do her regular blood glucose check. Vince felt motivated to manage his blood glucose because the device motivated him to check it. Pete was motivated by placing the device beside his bed. Serena and her son mentioned during the interview that Serena was more accountable to look after her blood glucose while using the telehealth device. Serena’s son stated that Serena was more aware of her blood glucose and food intake after using the device. Additionally, Vince, Heidi, Pete, and Ted also mentioned an improvement in awareness.

This telehealth device was not designed to motivate, build habits, or create awareness among patients. But this device did show the potential to change patients’ behavior if it had been integrated with persuasive technology strategies [23]. It could be improved by targeting specific behaviors, such as healthy eating habits [24] of T2D patients, to help manage their conditions better.

### Categorization of the Patients as Users

All patients did not use the device with the same degree of interest. We found different levels of interest in the patients based on the observations and their explanations during stages 1 and 2 of the CUE. Our persona categorization of the nine patients in the CUE includes enthusiastic, tolerant, indifferent, and resistant patients [6]. These categories need to be validated with a higher population of patients.

### Limitations of the Research

The CUE was conducted with a sample size of nine patients. To generalize these findings across the T2D population, future work should include a higher number of patients and expand quantitatively on findings of this research.

### Comparison With Prior Work

Most health researchers advocate larger, well-designed, controlled studies to gather evidence [25-27]. However, there is a gap. There are no studies that evaluate the effectiveness of telehealth in daily practice from patients’ lives; rather the studies strengthen current evidence [28]. This research is an approach to bridge that gap and increase evaluation of telehealth from a user (patient) perspective through CUE, unlike some recent usability studies with telehealth. For example, a study conducted for patients with T2D showed that usability improvements increased the acceptability by 57%, but studies of this sort are often explored to gather quantitative evidence only. They do not understand patients, unlike the CUE. To our knowledge, many studies conducted qualitative research as a component added onto a clinical trial, but no study has been conducted from ICT researchers from an HCI perspective that looks at telehealth and its impact on patients as users of these technologies. In another study, a 2016 investigation of patients with T2D who dropped out of an eHealth intervention used semistructured interviews to explore the reasons why patients opt out of a telehealth trial [29]. The CUE in this research used both contextual inquiry with semistructured interviews versus just semistructured interviews and uncovered both satisfied and dissatisfied patients [30].

Past qualitative work reported on telehealth-delivered educational interventions [31] and telephone interventions [31] did not improve medical conditions in T2D patients. Studies such as CUE can explore why some interventions worked or did not work. This kind of investigation had never been conducted in a clinical trial from an HCI perspective by ICT researchers. Generally, HCI evaluation is done during the development phase but, in this study, it was conducted in the rollout phase. Although domestication research had been undertaken with technology and users, domestication of a telehealth in-home monitoring device (in this case for T2D) has not been researched in the past until this study.

Another stream of studies took behavior change approaches in T2D management [32]. The CUE aligns more with this line of research. Researchers in the future should explore more in-depth into the role of the technology intervention and T2D management with approaches like CUE.

### Conclusions

Investigation of interactions between patients and a technology are critical in telehealth because it affects the overall outcome of a treatment. Disregard for the needs of patients, social and cultural habits, and the complex nature of health care systems results in relatively low impact and uptake of telehealth and eHealth technologies [33]. Some eHealth and telehealth interventions show dropout rates of up to 80% [34,35], but there is little knowledge about the UX-related dropouts. Therefore, we investigated a telehealth clinical trial through the HCI approach and investigated patients’ UX in a T2D clinical trial in Northern Queensland. We discovered that patients benefited from using the in-home monitoring device to manage their T2D regarding awareness, motivation, involvement, etc. Patients’ negative experiences with the technology—not all the patients engaged with the telehealth device equally—and design recommendations for future T2D telehealth were also found. We urge a global movement to advocate and practice HCI to complement all telehealth clinical trials and understand patients’ UX.

## Appendix

Multimedia Appendix 1 Contextual Design Process

## Abbreviations

CUE: Clinical User-experience Evaluation
HCI: human-computer interaction
ICT: information and communication technology
T2D: type 2 diabetes mellitus
UX: user experience
