# Supplementary material for: Complementing a Clinical Trial With Human-Computer Interaction: Patients’ User Experience With Telehealth
Source: JMIR Hum Factors. 2019 Jun 6;6(2):e9481. doi: 10.2196/humanfactors.9481 (PMC6592491; doi:10.2196/humanfactors.9481)
Supplement: Multimedia Appendix 1 [file humanfactors_v6i2e9481_app1.pdf]

Contextual Design

This file presents the contextual design work. Detailed steps of this work are presented at the end.

Users in the context of this paper were the patients. The contextual design process generally consists of eight steps: (1) Contextual Inquiry, (2) Interpretation Session, (3) Work Models and Affinity Diagramming, (4) Visioning, (5) Storyboarding, (6) User Environment Design, (7) Paper Mock-up, and (8) Interaction and Visual Design. The analysis in this paper covered the first three steps of the contextual design to create a UCD for T2D patients. A comparison of the current design of the technology used in this research with the UCD created through the first three steps of the contextual design is presented. The focus is predominantly on how the "system should have been" in contrast to "how the system is". Therefore, only steps 1 to 3 were appropriate to show the differences. Steps 4 to 8 generally are steps to create a new technology, thus not completed with the contextual design process.

Eight patients' (P1to P8) contextual inquiry field notes were used to create the UCD of this paper. The contextual inquiry of each patient was done in-situ in her/his own home. This study explored how the patients worked with the technology on a day-to-day basis. The contextual inquiry was conducted three months after the clinical trial to reduce any novelty effect. According to Beyer and Holtzblatt[42] users often cannot articulate *what* activity they do and *why* they do it, as work becomes very habitual. Hence, this research used contextual inquiry to understand the exact work process of the patients during a regular blood pressure and blood sugar upload session. Interpretation of the data was conducted after returning from the fieldwork.

The primary output of the contextual inquiry was work modelling for each user based on field notes kept by the researcher. These individual work models were consolidated so common patterns and structure could be seen without losing individual variations. The work modelling is represented using a *flow model*, a *sequence model* and *affinity diagramming*.

Flow Model

A flow model in contextual design captures communication and coordination between people to accomplish work. The nurses, doctors or any other professionals from whom the patients seek help were part of the formal workgroup while family

members are part of the informal workgroup. A workgroup simply means two or more individuals who interact with each other to work towards a purpose. Since, the coordination during this telehealth clinical trial is between the nurses and the patients, the doctor and family members are not considered as a formal work group. Any communication between the patients and any other human that is not part of the clinical trial was considered here as informal workgroup. The final consolidated flow model is presented in the following figure.

The Consolidated Flow Model

The ovals represent the humans, the rectangles represent the technologies and the arrows represent the flow of communication. Every patient has a doctor that they visit, generally every 3 months unless for urgent illnesses. The common workflow in the flow model of every patient was very similar. The flow model generally captures (i) every person that a user communicates with, and (ii) every technology (or artefact) a user uses, in the process of communication. A regular session entailed four steps where the patient would: (i) sit down in front of the technology; (ii) turn on the technology; (iii) check the schedule of blood glucose and blood pressure; and (iv) upload the blood glucose and blood pressure data using the glucometer and the sphygmomanometer. In addition, a patient could do the following activities (v) watch videos on the technology; (vi) accept videoconference requests and video conference (sent by nurses); read diabetes awareness information; and (vii) take a quiz to test one's diabetes awareness. However, only two patients (P1 Zach and P3 Yanicka) were seen to do the additional activities.

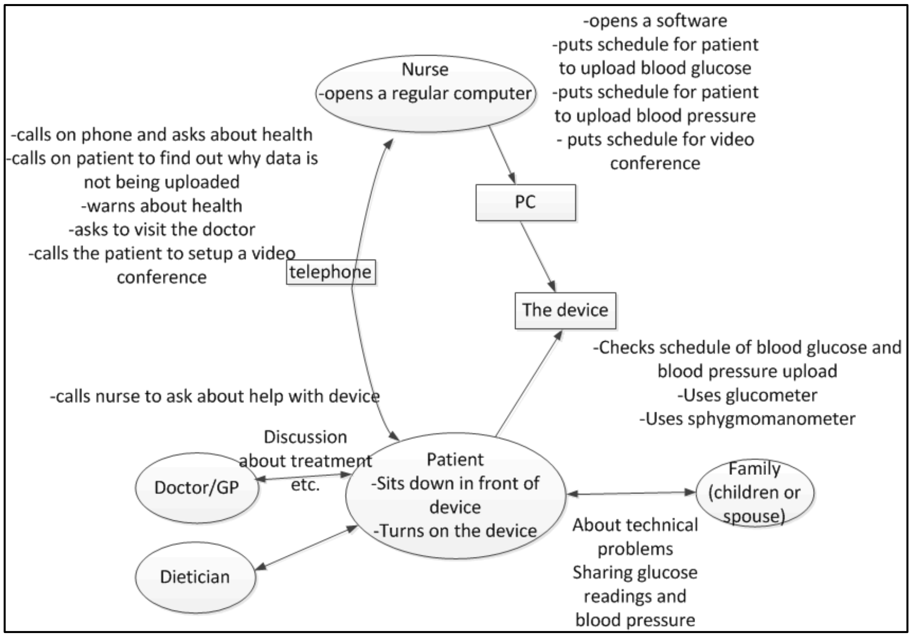

A nurse opened a regular computer in her office. Then, she could open the software that has patient information, view patient data, enter a schedule for the patient to upload blood glucose and blood pressure data and videoconference if needed. The patients and the nurses often engaged in verbal communication via telephone. All patients also communicated with their doctors during their physical visits. The technology in the clinical trial was not a mediator of doctor visits. Four patients (P4, P5, P6 and P7) communicated with some family members (spouse or children). Two patients (P8 and P5), additionally consult dieticians for better guidance on food choices.

Sequence Model

Sequence diagrams show the sequence of tasks carried out by a patient during the contextual inquiry. An intent in a sequence model shows the intention of the user, which leads to numerous steps that the user carries out to manifest that intent. A full sequence model of Zach (P1) is presented as a representative sequence model (Table 2) since Zach was one of the patients who accessed all functions; most of the other patients' sequence models were similar to Zach, but with fewer intents and triggers. A trigger shows the cause that leads a user to act out the intent. A break in a sequence is shown with the mark "~". A break interrupts the natural workflow; for example, when a user has to move away physically from the working technology or has to move out from the working application to a different application inside a system. All the other patients' sequence models are available online at the appendix of this multimedia appendix.

Breaks in sequence model

P1 (Zach) had three breaks in the sequence model (Table 2). The first break was caused right after Zach pricked his finger to get a blood drop. He immediately had to put a cotton bud on his fingertip with the other hand. Then he had to clean the other hand before he could tap on the touchscreen. When Zach wanted to find information about his medication, he had to go back to his laptop computer for two reasons (i) the name of the medicine was not listed in the technology and (ii) the technology does not have Internet browsing capability. This resulted in a third break in Zach's sequence model. All of the other seven patients experienced the first break, similar to Zach, to stop the bleeding followed by cleaning of the fingers on the other hand before they could tap on the touch screen. Not all patients kept records of their blood glucose and blood pressure data like Zach did. P7 (Uma), P4 (Bill), P5 (Serena) and P8 (Heidi) recorded their data in a diary. All of them also had a break similar to Zach's second break in their tasks. Patients who searched the Internet for additional data also had the same third break in the sequence as Zach.

Consolidated Sequence Model

The consolidation was done in multiple steps where the researcher generated each individual sequence diagram, then typed them in word processor, printed them, placed them altogether on a large work surface, consolidated them on an A3 size paper and finally translated them in a spreasheet.

	Activity	Intent	Abstract Step Strategy
1	Uploading Data using the device	To send data for nurse	Sits in front of the device
			Turns it on with the switch
			Waiting for auto log in
			Taps on the icon to see scheduled tasks
			Trigger: Device tells with voice message to measure blood glucose
			Opens the strip box
			Takes a strip
			Inserts strip in the glucometer
			Pricks finger with needle
			Puts blood drop on the strip
			Waits for the reply from the device
			The voice from device reads it out loud
			Puts the glucometer away
			Rubs his pricked finger with tissue paper
			Trigger: Device tells with voice message to measure blood pressure
			Wraps the cuff of the blood sphygmomanometer around the biceps of left arm
			Turns the button on sphygmomanometer
			Waits while the cuff tightens around the bicep
			Waits for the reply from the device
			The voice from device reads it out loud
			Takes off the hand out of the cuff and puts the sphygmomanometer aside

Figure A1. Consolidated sequence model- most used activity.

	Activity	Intent	Abstract Step Strategy
2	Watching Videos	Diabetes awareness	Taps on touchscreen button for informational videos

	Activity	Intent	Abstract Step Strategy
3	Reading diabetes education information	To be aware	Taps on touchscreen button for datasheet

	Activity	Intent	Abstract Step Strategy
4	Noting the data	To preserve record	P1 : Opens laptop and stores data in an excel sheet
			P3: Opens diary and writes down there
			P4: Writes down in diary

	Activity	Intent	Abstract Step Strategy
5	Looking at record	To compare today's data with another day's data	P1: Opens laptop and looks at excel sheet
			P3: Opens her diary and turns pages to look
			P4: Opens his diary and reads

	Activity	Intent	Abstract Step Strategy
6	Searching Information	Want to find out about some drug or situation not in the system	P1: Opens browser in laptop and searches<br>P3: Opens brower in mac book and searches

	Activity	Intent	Abstract Step Strategy
7	Troubleshoot	Trying to fix why someth is going wrong	Turn on off<br>Call Nurse via telephone<br>P6, P8 asks help from children

Figure A2. Consolidated sequence model- second to seventh activities.

A consolidated sequence model shows three columns - the leftmost shows the activity of the users, the middle shows the intent behind the activity and the rightmost shows the abstract step strategy taken by users. The order of presentation is not based on importance. The most common activity performed was uploading blood glucose and blood pressure data using the technology (Figure A1), since the technology had the main function of helping patients upload blood glucose data.

Only two patients P1 (Zach) and P3 (Yanicka) stated that they watched the awareness videos. The second and third activities (Figure A2) were to watch videos and read the information sheet, respectively. The fourth activity was to keep notes of the blood glucose and blood pressure data. The fifth activity was to look through the notes (self-created records) of the data and comparison. Both these activities are interrelated as one leads to doing the other. These activities (Figure A2) surfaced as very important according to the users. Patients performed this activity using their own artefacts because the current system does not have this function yet. Variations were seen among the three patients who did this activity. P1 (Zach) performed this activity with his own individual strategy by inputting the data in a Microsoft Excel spreadsheet on his laptop. P3 (Yanicka) performed this step by noting the data first on a piece of paper near the technology, which later she noted down in another diary. P4 (Vince) performed this step by putting the data directly in his diary. The sixth activity (Figure A2) is a rare activity that was performed by only two patients - P1 and P3. Both of them wanted to search for the information on their medication in the current system that the technology uses. The seventh activity is calling the nurse during troubleshooting of the technology. This was generally done by phone (Figure A2).

Affinity Diagramming

An affinity diagram brings together issues and insights about all users into a wall-sized hierarchical diagram [42].

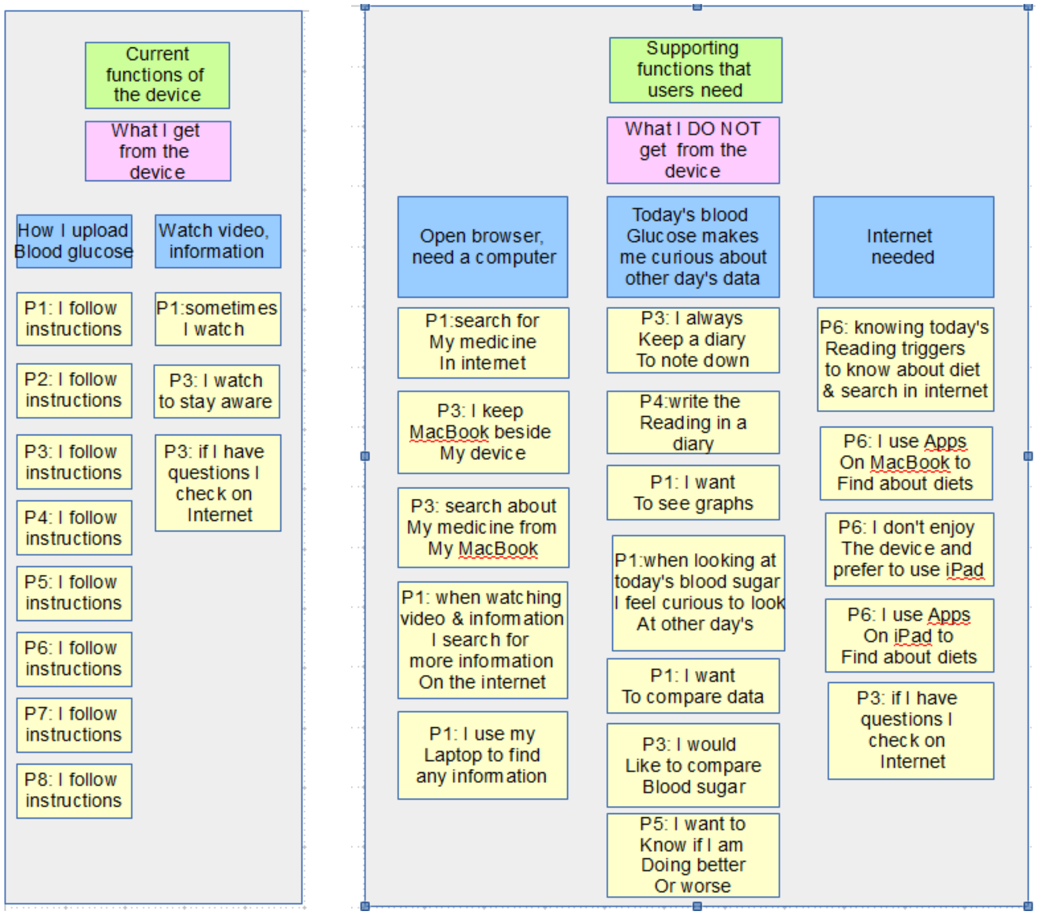

Figure A3. The complete affinity diagram.

Yellow represents the notes from individual patients of doing an activity. The activities in yellow are grouped together and the blue color code describes the general theme of the activities of the yellow notes under them. Pink represents another level above the blue level and abstracts the data further. Yellow, blue and pink are written in first person as if the user is directly speaking. The green labels of the affinity diagram (Figure A3) are the highest level of affinity. These represent activities of the patients' story. Figure A3 depicts main activities being performed

with the current in-home monitoring technology. These are represented with the green labels. They are: (1) Current function of the technology and (2) Supporting functions that users need. The leftmost green theme shows that the current function, which consists of users uploading their blood glucose, watching videos and accessing information sheet for awareness — are satisfying the users. The patients were performing these activities. However, the patients needed more things as part of their activities. These are joined under the green label. Figure 5 show the needs of the users. The patients need the following features as part of the activity: (i) need for Internet browser, (ii) ability to store, view and compare blood glucose of different days, and (iii) Internet connectivity to find information.
